# Fluoxetine for the treatment of onychotillomania associated with obsessive–compulsive disorder: a case report

**DOI:** 10.1186/s13256-022-03652-9

**Published:** 2022-11-20

**Authors:** Sumayah Aljhani

**Affiliations:** Department of Psychiatry, College of Medicine, Qassim University, Qassim, Saudi Arabia

**Keywords:** Onychotillomania, Obsessive–compulsive disorder, Focused body repetitive behavior, Nail tic disorders, Paroxetine, Fluoxetine

## Abstract

**Background:**

Obsessive–compulsive disorder is a condition in which patients experience an obsession and/or a compulsion. It has a high impact on the quality of life, and is associated with an increased prevalence of psychiatric comorbidities in patients. Onychotillomania is an underestimated psychodermatosis caused by repeated self-inflicted damage to the nail unit. In patients, it is characterized by an obsessive or irrepressible impulse to repeatedly damage their own nails, resulting in their destruction. It is a chronic condition that is difficult to manage, largely because of its psychocutaneous character, as well as its high tendency to interact with underlying neuropsychiatric diseases or other behavioral disorders. Only a few studies have reported an association between obsessive–compulsive disorder and onychotillomania, which typically presents with therapeutic challenges. Cognitive behavioral therapy, physical-barrier approaches, and pharmaceutical treatments have been reported to be beneficial in the management of onychotillomania; however, no major clinical studies have investigated the effectiveness of these therapies. Onychotillomania remains a clinical and therapeutic issue owing to the lack of evidence-based treatment techniques.

**Case presentation:**

We report a case of an 18-year-old, middle-eastern female patient who developed onychotillomania when she was being treated with paroxetine for obsessive–compulsive disorder and was showing partial improvement. The patient developed side effects from paroxetine, and was switched to fluoxetine. Thereafter, improvement in her obsessive–compulsive disorder was observed, which relapsed when treatment was discontinued. However, the onychotillomania symptoms did not reemerge.

**Conclusion:**

Onychotillomania typically presents both diagnostic and therapeutic challenges. Fluoxetine plays an important role in the treatment of onychotillomania and other psychiatric disorders. However, large-scale studies should be conducted before these outcomes can be generalized.

## Background

Obsessive–compulsive disorder (OCD) is a condition in which patients experience persistent unwanted thoughts, ideas, or feelings (obsessions), which triggers them to perform an action repeatedly (compulsions), such as washing hands, checking objects, or cleaning, which may interfere with everyday tasks and interpersonal relationships [1]. OCD has a high impact on the patient’s quality of live, and has been associated with an enhanced prevalence of psychiatric comorbidities [2], as well as dermatological disorders such as cellulitis [3]. OCD is widely prevalent in adults, adolescents, and children worldwide [4]. In most cases, the disease is detected by the age of 19 years, and it occurs earlier in males than in females [5].

Onychotillomania is an unusual and often misunderstood behavioral tendency that affects the nail apparatus. Patients are identified by an obsessive or irrepressible impulse to repeatedly damage their nails, either with their fingers or with other objects, resulting in obvious and even irreparable self-destruction of the nail unit [6]. A person with this impulse may be conscious or unconscious of its appearance [7, 8]. Regarding clinical manifestations, onychotillomania is characterized by various nonspecific findings such as unusual nail morphology, nail plate injury, and periungual skin irritation [9]. Furthermore, characteristics that are not present in other nail disorders, such as loss of the nail plate, presence of several obliquely directed nail bed hemorrhages, gray coloration of the nail plate, and wavy lines, may also be observed [10]. It affects 0.9% of the global population, and can cause permanent nail deformation, melanonychia, and infections [11]. Onychotillomania diagnosis requires an understanding of the patient’s clinical history, as treatment methods may include behavioral treatments and psychiatric drugs [9].

Approximately 33% of individuals with dermatological disorders are affected by emotional and psychological variables [12]. It is possible that a mental illness may be caused by an underlying dermatological problem, as in the case of onychotillomania [7]. Few studies have reported an association between OCD and onychotillomania [13, 14]. In psychiatry, onychotillomania is considered an impulse control disorder, similar to OCD, compulsive gambling, and kleptomania [7]. It has also been categorized as a habitual deformity that may develop due to mental and emotional stress, or as a type of OCD [15]. Although onychotillomania was not listed in the Diagnostic and Statistical Manual of Mental disorders-5 or the International Classification of Diseases-10 as a separate disorder, it can be considered as a body-focused repetitive behavioral disorder that includes other traits, such as nail biting and cheek chewing. As onychotillomania is often associated with serious depression and OCD, a correct diagnosis of the condition is crucial but difficult. Importantly, onychotillomania may also indicate the presence of other mental problems such as OCD [16] and depression [17].

Individuals suffering from onychotillomania should consider seeking treatment for any underlying psychological illnesses. In addition to onychotillomania, onychophagia and trichotillomania-like symptoms are among the most prevalent dermatological manifestations of OCD [18]. In a study involving 509 patients with OCD, 56 patients had nail-biting habits, which is substantially higher than that in the general population [19]. However, as hypothesized, OCD is not the underlying psychopathology for every patient with onychotillomania. Before concluding that a patient with onychotillomania has OCD, it is necessary to rule out alternative psychiatric diagnoses, such as delusions or simple habit disorders [7].

After psychiatric evaluation and diagnosis, it is critical that the underlying mental disease be identified and treated with psychotropic medications [20]. Individual psychotherapy and behavioral therapy are the two most commonly used therapies for OCD. Additionally, oral drugs are often used to treat OCD.

Onychotillomania typically presents with therapeutic problems. It is a chronic and difficult-to-manage condition, largely because of it has a psychocutaneous character as well as a high tendency to interact with underlying neuropsychiatric diseases or other behavioral disorders [8]. Reports have shown that cognitive behavioral therapy, physical-barrier approaches, and pharmaceutical treatments have some advantages, although no major clinical studies have investigated the effectiveness of these therapies. Onychotillomania remains a clinical and therapeutic issue for dermatologists, pediatricians, internists, and psychiatrists in practice because there are no evidence-based treatment techniques [11].

Various methods have been reported for treating onychotillomania. Nonpharmacological treatments include covering the nails or toes with physical barriers, such as an Unna boot [7, 21], gloves, or bandages [22], and cyanoacrylate adhesive [23]. Psychotherapy is also used and is mainly directed towards performing a competing response, such as gripping and pulling the hands [24], or acceptance-enhanced behavior therapy, including habit reversal and stimulus control. [25]

The effectiveness of pharmacological treatments, consisting of the administration of various drugs such as *N*-acetylcysteine [26, 27], intravenous triamcinolone acetonide followed by a topical combination of calcipotriol and betamethasone dipropionate [6], citalopram, and zolpidem hemitartrate [28], as well as behavioral treatment [25] have been reported for onychotillomania.

## Case presentation

Here we present a case of a middle-eastern female patient with OCD who developed onychotillomania at the age of 17 years. The patient’s OCD condition initially improved with fluoxetine treatment but relapsed later. However, onychotillomania, which developed as a consequence of OCD, did not reemerge. The patient was a healthy Middle Eastern teenage female who developed OCD around the age of 16 years, precipitated by a stressful event. The patient experienced recurrent, intrusive, distressing, and time-consuming obsessional thoughts of being surveilled through her phone. These obsessions also made her functionally impaired. Consequently, the patient started to avoid using the phone and eventually damaged it. Nevertheless, her symptoms persisted even with a new phone. Furthermore, the patient was worried about harm to her family, although this symptom did not qualify as an anxiety disorder or OCD. The patient had no other symptoms of OCD, nor did her symptoms meet the criteria for psychotic disorders. She was under the care of another physician at the time of onset of the condition, and was treated with fluoxetine, which was changed to paroxetine with clear justification.

**Fig. 1:**
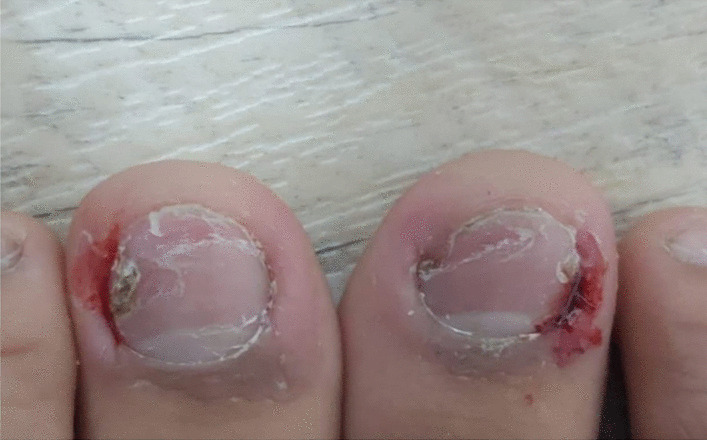
Lateral erythematous, swollen, and bleeding nail folds of the big toes

**Fig. 2:**
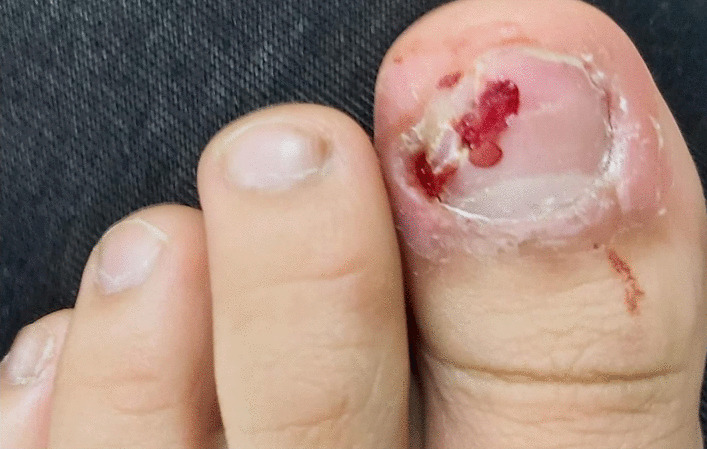
Distolateral swelling and bleeding indicating ingrowing of the left big toenail

The patient commenced paroxetine 20 mg titrated to 30 mg daily and started showing improvements. However, 5 months later, her OCD symptoms relapsed with depressive features and floating passive suicide ideation. Additionally, she demonstrated nail picking that was mainly directed towards the big toes and the skin around the nails using only her fingers. On examination, the lateral nail fold was erythematous and swollen with bleeding in both big toes, which may indicate a distolateral ingrowth and the nails showed scales and signs of distal nail tearing by the patient. Sometimes the patient’s injuries required bandaging (Figs. 1 and 2). These symptoms were distressing, occurred daily, and were preceded by an urge and followed by a relief. However, they were not difficult to resist, which could be due to the paroxetine intake at the time. This complaint was not related to anxiety or depressive symptoms, and the patient had no other body-focused repetitive behaviors. Subsequently, the dose of paroxetine was increased to 40 mg. Two months later, the patient started to experience side effects that included weight gain and difficulty in urination.

Medication was switched to fluoxetine at a dose of 20 mg titrated, which was later increased to 40 mg daily. After the resolution of OCD and nail-picking symptoms, upon the patient’s request, 6–7 months later, the medication was reduced. The fluoxetine dose was tapered and discontinued over 3 months without any relapse in symptoms. However, 2–3 months later, the OCD symptoms relapsed and treatment with fluoxetine 20 mg was recommenced. Importantly, the onychotillomania symptoms did not reappear. At the time of writing this report, approximately 2 months after restarting fluoxetine, the patient’s OCD had partially improved and onychotillomania remained remitted.

## Discussion

Onychotillomania is an underestimated psychodermatosis caused by repetitive self-inflicted damage to the nail unit. The condition manifests with minor-to-severe nail plate morphologies [6]. It most often occurs in young people who are otherwise mentally normal, but it may also be related to anxiety in rare cases. Onychotillomania has been reported to occur with other psychiatric illnesses such as depression anxiety, psychosis, dissociation, and dermatitis artefacta [29].Occasionally, it may manifest as a symptom of OCD in extreme circumstances [14]. However, onychotillomania has a greater tendency to be connected with basic psychiatric comorbidities, such as fixated hypochondriacal delusions, anxiety, and OCD as compared with onychophagia [13, 23, 30], which the later defined as chronic chewing of the cuticle, nail bed, nail folds, or nail plate [29].

Despite the severity of the condition, only a few studies exist on onychotillomania, with the majority of them highlighting the importance of a thorough assessment and treatment of the comorbid conditions such as OCD, depression, and tic. In the current case, the patient had OCD associated with onychotillomania, and was treated for OCD with paroxetine, and partial improvement was observed. However, because the patient suffered from paroxetine side effects, she was switched to fluoxetine. Thereafter, improvement was observed in OCD, which later relapsed, but the symptoms of onychotillomania did not reappear. This case demonstrates the effectiveness of fluoxetine in treating onychotillomania. The effectiveness of higher doses of fluoxetine in treating onychotillomania has previously been reported [14]. Fluoxetine stops nail picking in adult males at only 20 mg [15]. However, in this case, onychotillomania remitted at a relatively small dose and did not relapse even after the discontinuation of medication despite the OCD relapse. Another study reported ceasing of nail picking with sertraline [30] and reduction of nail and cuticle picking with pimozide [31] in adult patients. In contrast, selective serotonin reuptake inhibitors (SSRIs) did not influence nail picking in another case [25].

It is necessary to comprehensively assess the patient’s mental health before choosing the appropriate treatment. Although evidence-based research to support their usefulness and effectiveness is lacking, nail tic disorders have been treated using various methods. Although SSRIs have been beneficial in treating severe onychotillomania, some patients with comorbid psychiatric illnesses develop mania as a side effect of this treatment. Therefore, it is essential to thoroughly evaluate the patient to determine the disorder intensity and any accompanying mental disease and tailor the therapy accordingly.

Other nonpharmacological techniques have been tested, including psychotherapy, hypnosis, cue-controlled relaxation, and various behavioral therapies. There is substantial level 2 evidence supporting the use of behavioral therapy as the first-line treatment option [32]. Additionally, the regular use of unpleasant topical preparations on the nails and periungual areas is encouraged as it can deter patients from biting their nails [14]. Occlusive coverings such as gloves, dressings, and sticky tape as shields between the mouth and fingers are also frequently used to prevent the nails from being damaged by the trauma [11, 16, 30].

The effectiveness of fluoxetine has been reported in skin picking disorders alone as well as in combination with psychiatric disorders, such as OCD [33–36]. The use and effectiveness of fluoxetine has also been reported for various psychiatric disorders, including OCD [37–41]. However, to the best of our knowledge, this is the first case report of OCD relapse after treatment with fluoxetine, where the symptoms of onychotillomania did not re-appear.

## Conclusion

Onychotillomania typically presents both diagnostic and therapeutic challenges. It is a chronic and difficult-to-manage condition, largely because it has a psychocutaneous character and a high tendency to interact with the underlying neuropsychiatric diseases or other behavioral disorders. In this report, fluoxetine had an advantageous effect in treating onychotillomania. However, this conclusion is based on a time-limited clinical observation of a single patient and, therefore, large-scale studies should be conducted around pharmacological treatments to generalize these outcomes.

## Data Availability

All data related to the study are presented in this report. Any further details or inquiries will be available upon request from the corresponding author.

## Abbreviations

OCD: Obsessive–compulsive disorder
SSRIs: Selective serotonin reuptake inhibitors
